# Supplementary material for: Ferri- and ferro-electric switching in spontaneously chiral polar liquid crystals
Source: Nat Commun. 2025 Aug 13;16:7510. doi: 10.1038/s41467-025-62684-z (PMC12350638; doi:10.1038/s41467-025-62684-z)
Supplement: Supplementary file 1 — Supplementary Information [file 41467_2025_62684_MOESM1_ESM.pdf]

Ferri- and Ferro-Electric Switching in Spontaneously Chiral Polar Liquid Crystals

Supplementary information

Jordan Hobbs[*1], Calum J. Gibb[2], and Richard. J. Mandle[1,2]

[1] School of Physics and Astronomy, University of Leeds, Leeds, UK, LS2 9JT
[2] School of Chemistry, University of Leeds, Leeds, UK, LS2 9JT

*Author for correspondence e-mail: j.l.hobbs@leeds.ac.uk

Contents

1. Supplementary methods
2. Supplementary results
3. Organic synthesis
4. Supplementary References

1. Supplementary Methods

1.1. Chemical Synthesis

Chemicals were purchased from commercial suppliers (Fluorochem, Merck, ChemScene, Ambeed) and used as received. Solvents were purchased from Merck and used without further purification. Reactions were performed in standard laboratory glassware at ambient temperature and atmosphere and were monitored by TLC with an appropriate eluent and visualised with 254 nm light. Chromatographic purification was performed using a Combiflash NextGen 300+ System (Teledyne Isco) with a silica gel stationary phase and a hexane/ethyl acetate gradient as the mobile phase, with detection made in the 200-800 nm range. Chromatographed materials were filtered through 200 nm PTFE frits and then subjected to re-crystallisation from an appropriate solvent system.

1.2. Chemical Characterisation

Chemical materials were characterised by NMR spectroscopy using a Bruker Avance III HDNMR spectrometer operating at 400 MHz, 100.5 MHz or 376.4 MHz (^{1}H, ^{13}C{^{1}H} and ^{19}F, respectively). High resolution mass spectrometry data (HRMS) was collected using a Bruker MaXis Impact spectrometer with a negative ESI source (VIP-HESI); the sample was introduced via direct infusion as solution in MeCN. HPLC analysis was performed using an Agilent 1290 Infinity II system fitted with a poroshell 120 ec-c18 column running a water:MeCN gradient.

1.3. Thermal Analysis

Differential scanning calorimetry (DSC) measurements were performed using a TA Instruments Q2000 DSC instrument (TA Instruments, Wilmslow UK), equipped with a RCS90 Refrigerated cooling system (TA Instruments, Wilmslow UK). The instrument was calibrated against an Indium standard, and data were processed using TA Instruments Universal Analysis Software. Samples were analysed under a nitrogen atmosphere, in hermetically sealed aluminium TZero crucibles (TA Instruments, Wilmslow, UK) and subjected to three analysis cycles. In all cases, samples were subject to heating and cooling at a rate of 10 K min^{-1}. Phase transition temperatures were measured as onset values on cooling cycles for consistency between monotropic and enantiotropic phase transitions, while crystal melts were obtained as onset values on heating.

1.3. Optical Microscopy

Polarised light optical microscopy (POM) was performed using a Leica DM2700P polarised light microscope (Leica Microsystems (UK) Ltd., Milton Keynes, UK), equipped with 10x and 50x magnification, and a rotatable stage. A Linkam TMS 92 heating stage (Linkam Scientific Instruments Ltd., Redhill, UK) was used for temperature control, and samples were studied sandwiched between two untreated glass coverslips. Images were recorded using a Nikon D3500 Digital Camera (Nikon UK Ltd., Surbiton, UK), using DigiCamControl software.

1.4. X-ray Scattering

X-ray scattering measurements, both small angle (SAXS) and wide angle (WAXS) were recorded using an Anton Paar SAXSpoint 5.0 beamline machine. This was equipped with a primux 100 Cu X-ray source with a 2D EIGER2 R detector. The X-rays had a wavelength of 0.154 nm. Samples were filled into either thin-walled quartz capillaries or held between Kapton tape. Temperature was controlled using an Anton Paar heated sampler with a range of -10 °C to 120°C and the samples held in a chamber with an atmospheric pressure of <1 mbar. Samples were held at 120°C to allow for temperature equilibration across the sample and then slowly cooled while stopping to record the scattering patterns. Background scattering off the sample holders was subtracted from the obtained patterns after being appropriately scaled.

The samples were not formally aligned and so these measurements can be considered as "powder" samples. It should be noted that some spontaneous alignment of the LCs both within the capillaries and between the Kapton tape did occur leading to the classic "lobe" pattern seen in the 2D patterns. 1D patterns were obtained by radially integrating the 2D SAXS patterns. Peak position and FWHM was recorded and then converted into d spacing following Bragg's law. In the tilted smectic phases, the tilt was obtained from:

$$\frac{d_c}{d_A} = \cos\theta \qquad \textbf{(1)}$$

where d_c is the layer spacing in the tilted phase, d_A is the extrapolated spacing from the non-tilted preceding phase, extrapolated to account for the weak temperature dependence of the preceding phases due to shifts in conformation and order, and θ the structural tilt angle.

1.5. Measurement of Spontaneous Polarization (P_S)

Spontaneous polarisation measurements are undertaken using the current reversal technique. Triangular waveform AC voltages are applied to the sample cells with an Agilent 33220A signal generator (Keysight Technologies), and the resulting current outflow is passed through a current-to-voltage amplifier and recorded on a RIGOL DHO4204 high-resolution oscilloscope (Telonic Instruments Ltd, UK). Heating and cooling of the samples during these measurements is achieved with an Instec HCS402 hot stage controlled to 10 mK stability by an Instec mK1000 temperature controller. The LC samples are held in 4μm thick cells with no alignment layer, supplied by Instec. The measurements consist of cooling the sample at a rate of 1 Kmin^{-1} and applying a set voltage at a frequency of 20 Hz to the sample every 1 K. The voltage was set such that it would saturate the measured P_S and was determined before final data collection.

There are three contributions to the measured current trace: accumulation of charge in the cell (I_c), ion flow (I_i), and the current flow due to polarisation reversal (I_p). To obtain a P_S value, we extract the latter, which manifests as one or multiple peaks in the current flow, and integrate as:

$$P_S = \int \frac{I_p}{2A} dt \qquad \textbf{(2)}$$

where A is the active electrode area of the sample cell.

1.6. Birefringence Measurements

Birefringence was measured using a Berek compensator mounted in a Leica DM 2700 P polarised optical microscope. The birefringence was measured in the centre of the active ITO area for the IPS cells used here. Error bars were assessed by repeating the measurement several times and using the average to obtain a statistical error.

1.7. Transmission Spectra

A microscope equipped coupled to an Avantes AvaSpec-2048 XL spectrometer which used to record transmission spectra of samples. The rubbing direction was set parallel to the light polarization direction, but no analyzer was inserted. A spectrum from the phase above the spontaneously chiral phases was used as the reference state of each sample

1.8. Pitch Measurements

The pitch was measured by illumination of the sample from the bottom via a 405 nm laser. The angle of diffraction was measured on a flat screen set 1.8cm away from the sample.

2. **Supplementary Results**

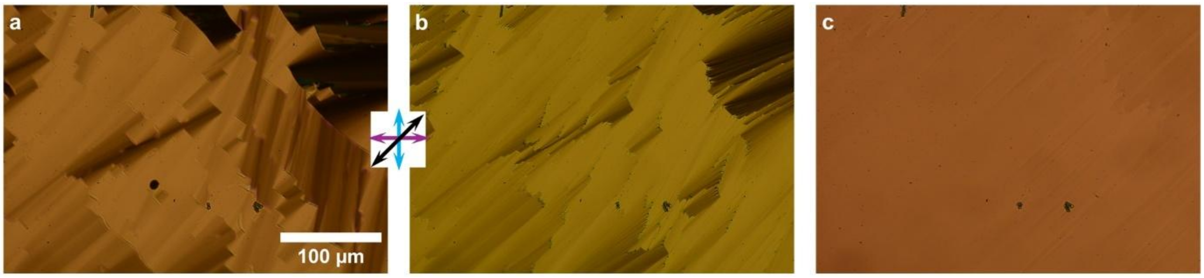

Fig. S1 POM images of compound **1** in a 5 µm thick parallel rubbed planar cell at a) 120 °C in the SmA_F phase on heating, b) 150 °C in the SmA phase on heating and c) 120 °C in the SmA_F phase on cooling. The blue and purple arrows indicate the polariser axes while the black arrow indicates the rubbing direction.

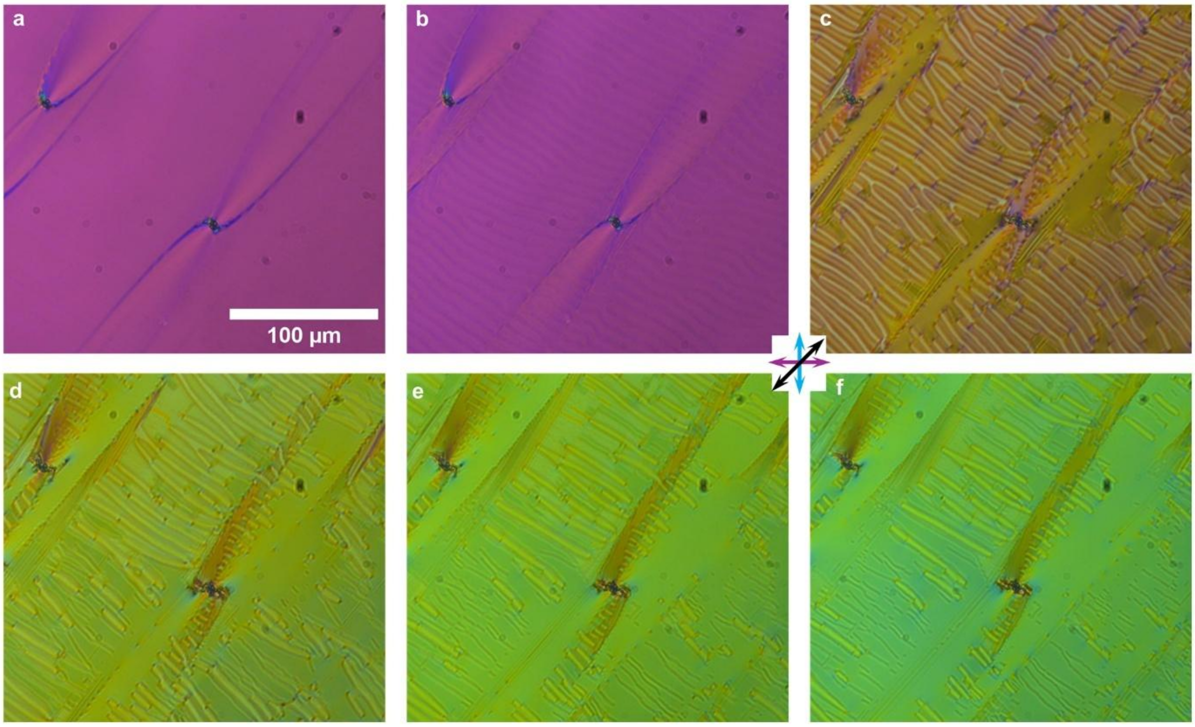

Fig. S2 POM images of compound **2** in a 5 µm thick parallel rubbed planar cell at a) 105 °C in the N_F phase on cooling and b) 104 °C, c) 103 °C, d) 102 °C e) 101 °C and f) 100 °C in the N_{TBF} phase on cooling. The blue and purple arrows indicate the polariser axes while the black arrow indicates the rubbing direction.

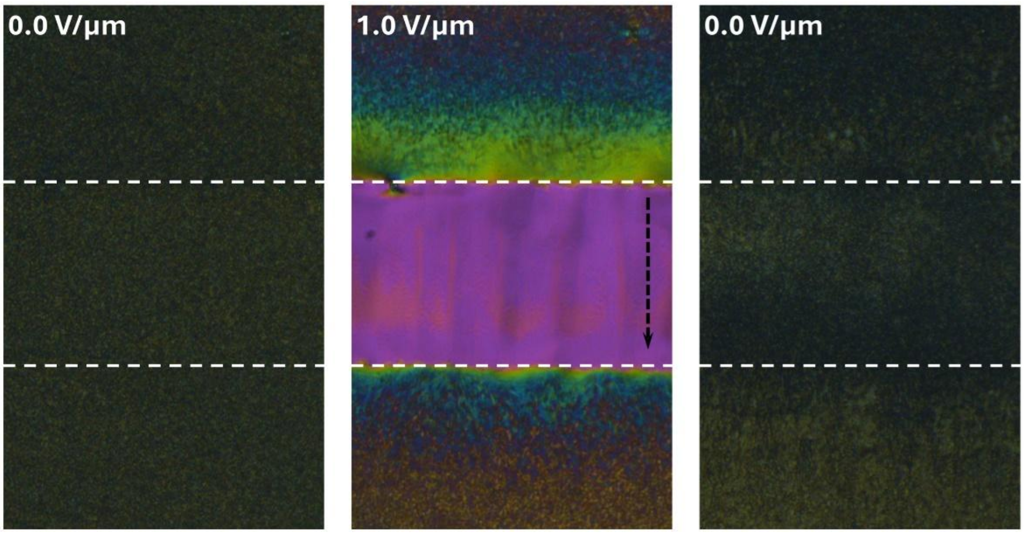

Fig. S3 POM observations of compound **2** in the SmC_P^H phase at 92 ºC in a 5.5 μm parallel rubbed planar aligned cell with a pair of in plane electrodes separated by 100 μm. Voltage is applied OFF-ON-OFF.

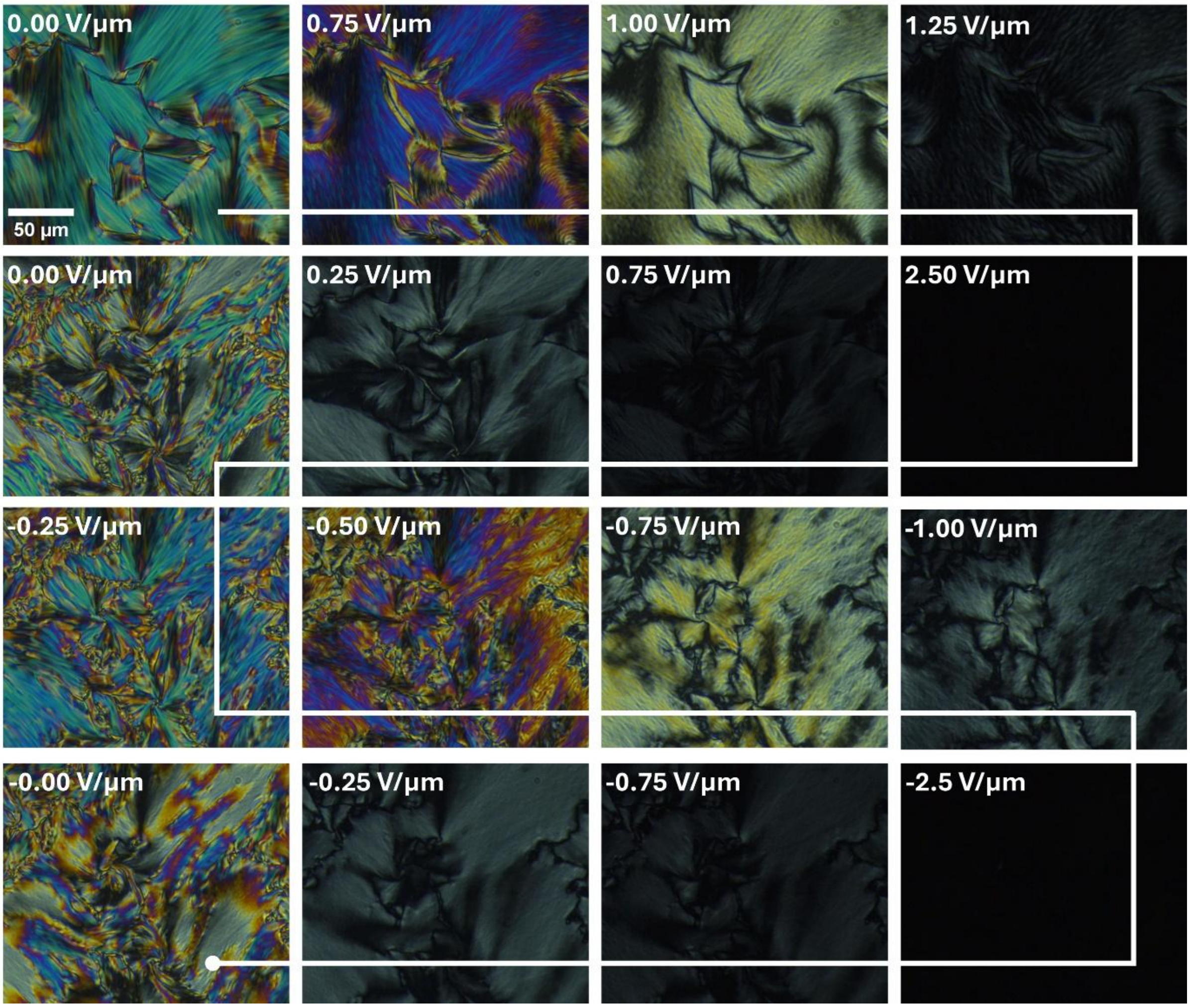

Fig. S4 POM observations of compound **1** in the SmC_P^H phase at 70 ºC in a 4 μm cell with no alignment layer. Field strength values indicate the strength of DC field applied across the cell gap.

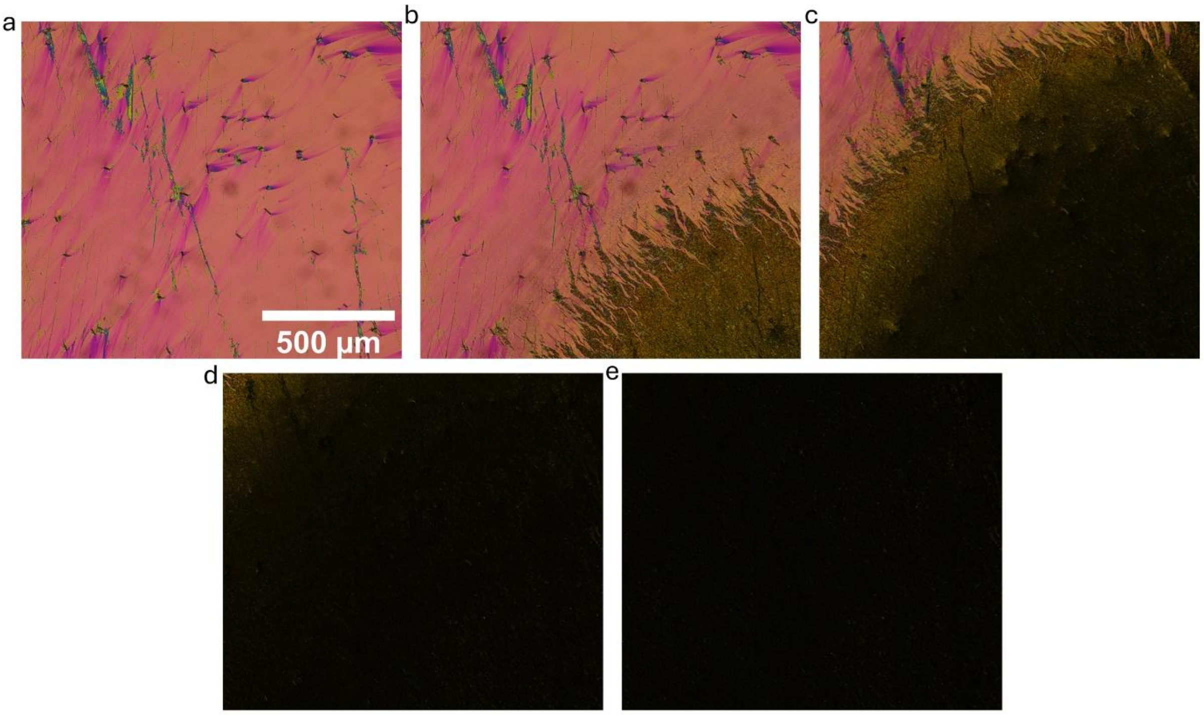

Fig. S5 POM observations of **3** at the boundary between the N_F and SmC_P^H phases over 1 °C showing the slow growth and dendritic tendrils.

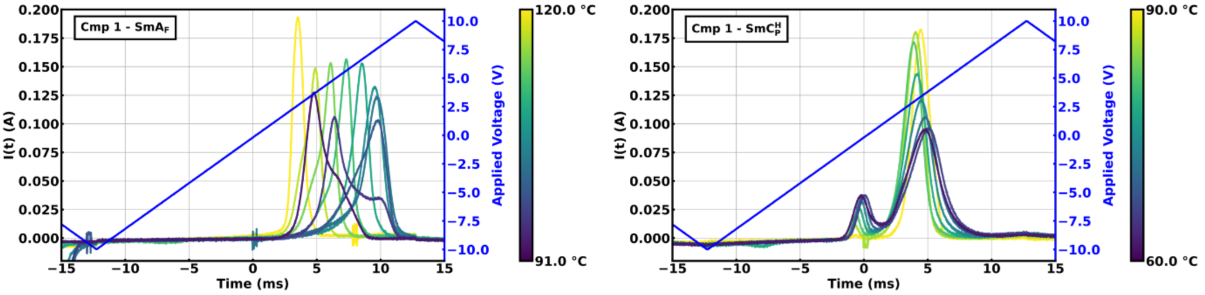

Fig. S6 Current responses for compound **1** in a 4 µm cell with no alignment layer. The applied field is a triangle wave with 10 V (2.5 V/µm) at 10 Hz.

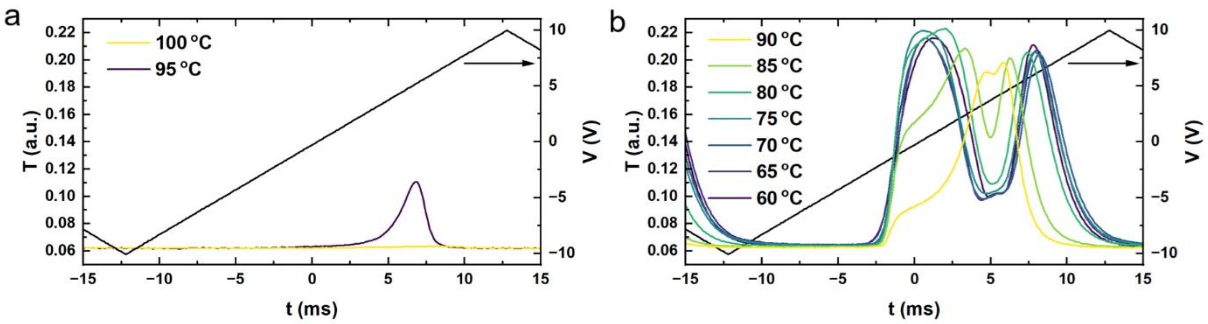

Fig. S7 Electro-optic response for compound **1** in a 4 µm cell with no alignment layer set between crossed polarisers. The applied field is a triangle wave with 10 V (2.5 V/µm) at 10 Hz.

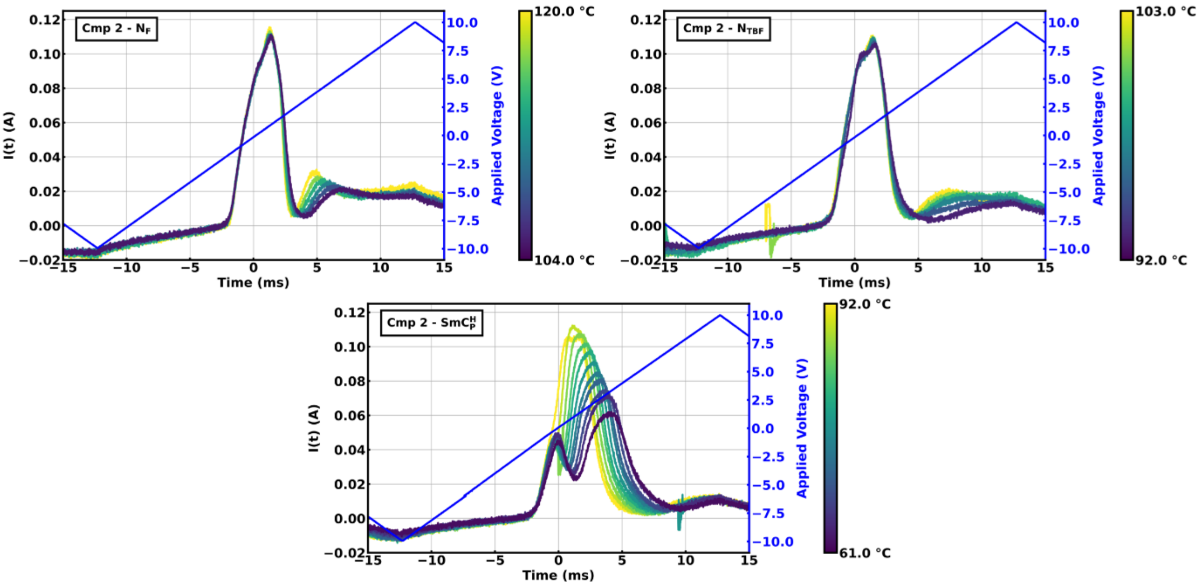

Fig. S8 Current responses for compound **2** in a 4 μm cell with no alignment layer. The applied field is a triangle wave with 10 V (2.5 V/μm) at 10 Hz

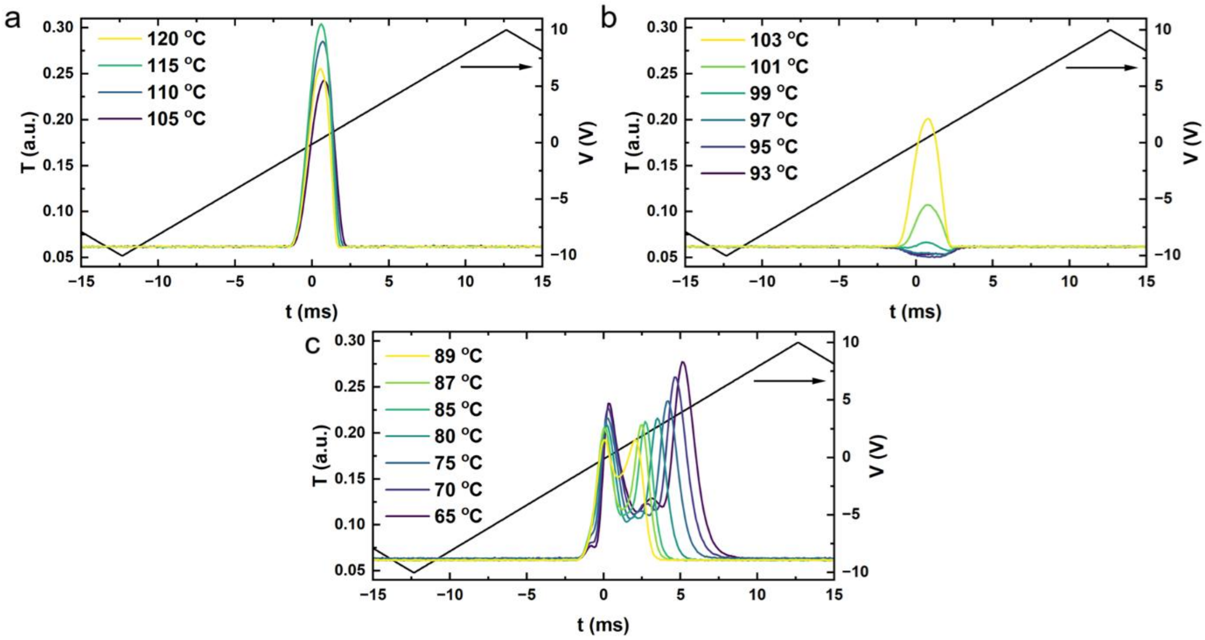

Fig. S9 Electro-optic response for compound **2** in a 4 μm cell with no alignment layer set between crossed polarisers. The applied field is a triangle wave with 10 V (2.5 V/μm) at 10 Hz.

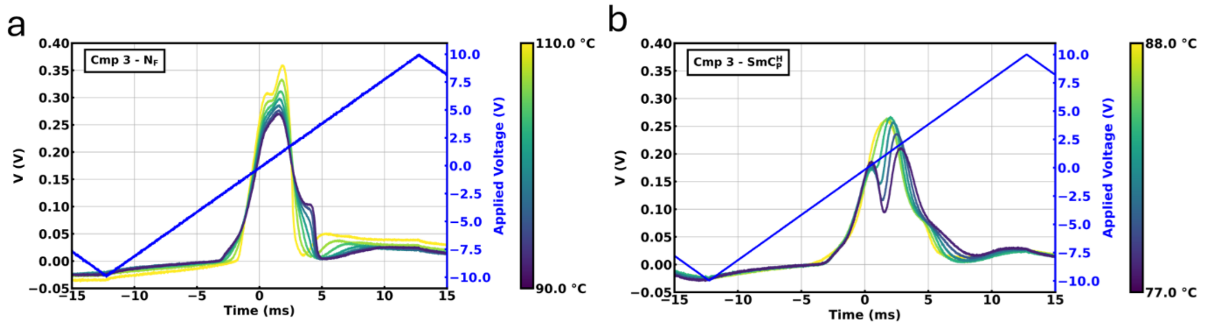

Fig. S10 Current responses for compound **3** in a 4 µm cell with no alignment layer. The applied field is a triangle wave with 10 V (2.5 V/µm) at 10 Hz

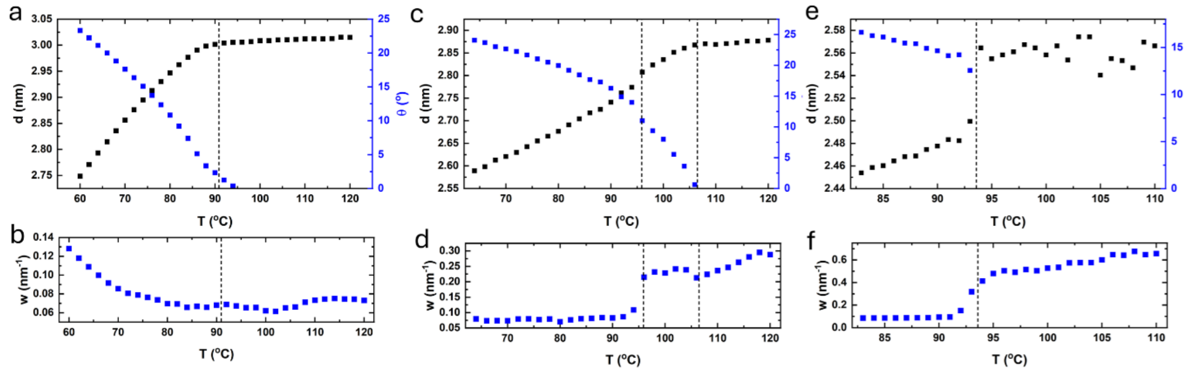

Fig. S11 Temperature dependence and FWHM of the small angle scattering peaks of a,b) **1**, c,d) **2** and e,f) **3.**

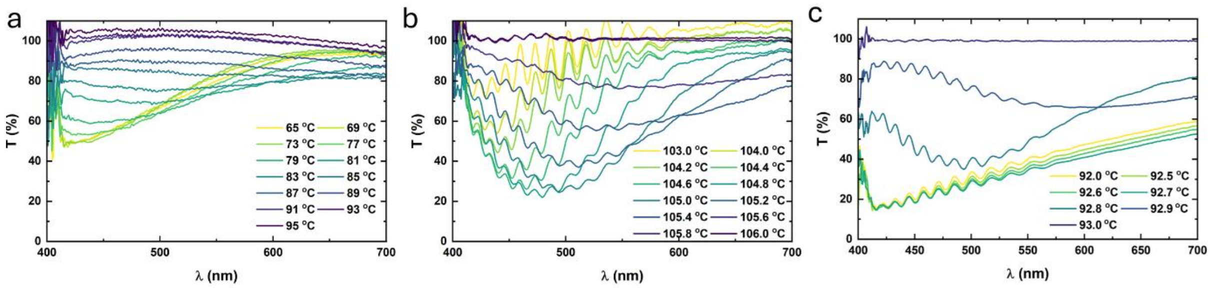

Fig. S12 Transmission spectrum of a) **1** b) **2** and c) **3**. For all samples the wavelength shifts to shorted wavelengths.

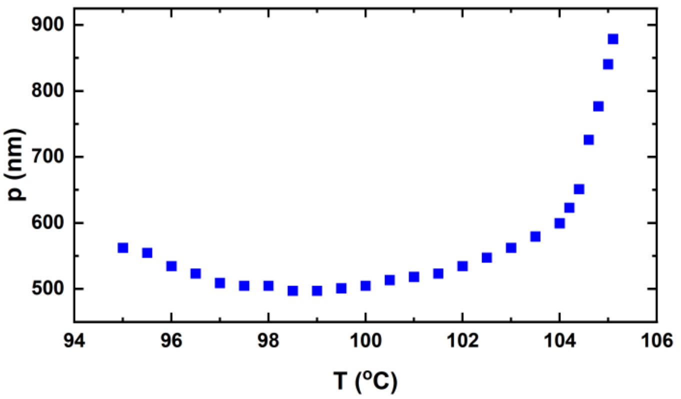

Fig. S13 Temperature dependence of **2** in the N_{TBF} phase obtained on cooling in a 5 μm parallel rubbed planar cell.

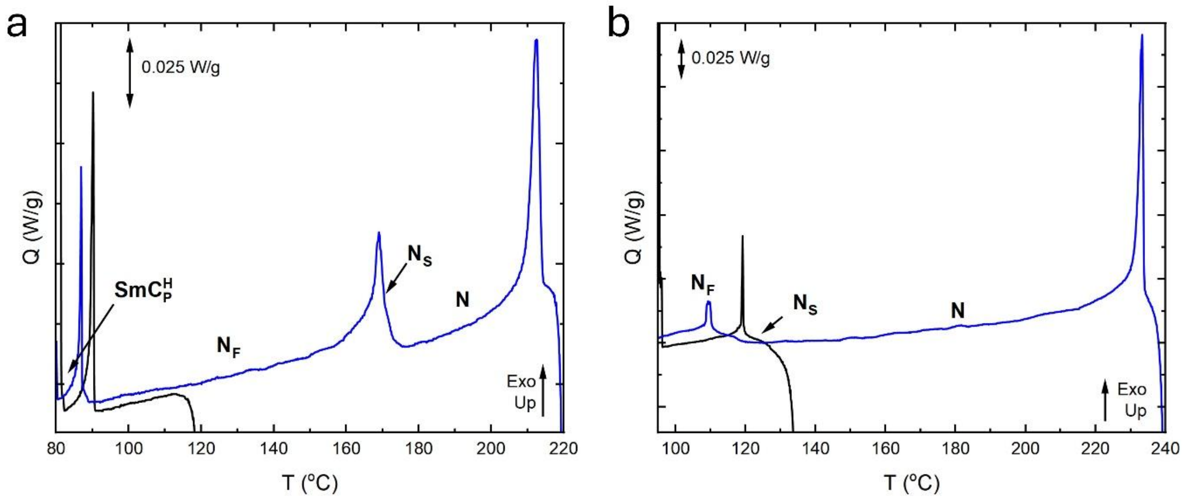

Fig. S14 DSC scans for a) **3** and b) **4** showing two cycles. The first (black) is around a chemically stable point to obtain the most accurate transition temperatures for the transitions we can see in that winder. The second cycle (blue) goes to higher temperatures where degradation of the lower temperature transitions can be observed, but this is necessary to observe and measure the higher temperature transitions.

3 Organic Synthesis

The synthetic route and chemical characterisation data associated with intermediate products and materials **2-4** is given below. The synthesis of material **1** and the preparation of 2,6-difluoro-4-(5-propyl-1,3-dioxan-2-yl)benzoic acid is reported elsewhere [1,2]

3.1 General Suzuki Coupling Protocol

A reaction flask was charged with 5-bromo-2-(difluoro(3,4,5-trifluorophenoxy)methyl)-1,3-difluorobenzene (20 mmol) and the relevant phenol boronic ester (21 mmol) which were dissolved in 150 mL of THF and 60 mL of 2M K_2CO_3(aq). The resultant solution was sparged with $N_{2(g)}$ for 20 mins. In a separate vial, 5 mL of THF was sparged with $N_{2(g)}$ for 15 minutes before Pd(OAc)2 (50 mg) and SPhos (100 mg) were added and stirred for a further 5 mins. The reaction flask was then heated to 70 ℃ and the catalyst solution added in one portion. The reaction was monitored by TLC with the completion of the reaction being determined by the complete consumption of the bromo-sub-straight. The reaction was then cooled, the aqueous and organic layers separated with the organics being dried over $MgSO_4$. The organics were then passed through a silica plug before the filtrate was concentrated under reduced pressure and purified by flash chromatography over silica gel with a gradient of hexane/ethyl acetate using a Combiflash NextGen300+ system using a gradient elution from hexane - ethyl acetate. Finally, the product re-crystallised from hexane:toluene (10:1) to give the appropriate phenols.

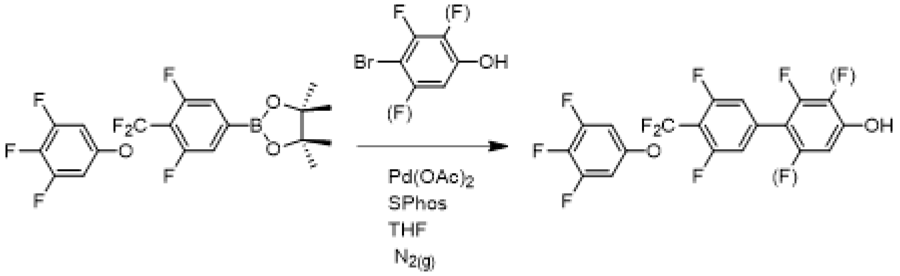

Scheme 1. General Suzuki coupling protocol.

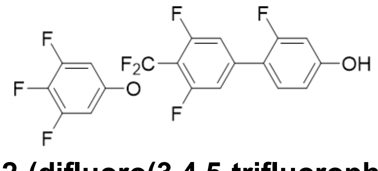

5-(2-fluoro-4-hydroxyphenyl)-2-(difluoro(3,4,5-trifluorophenoxy)methyl)-1,3-difluorobenzene

Yield: (off-white powder) 7.06 g, 84 %

R_f (DCM): 0.35

^{1}H NMR (400 MHz, DMSO) (δ): 10.38 (s, 1H, Ar-O**H**), 7.50 (t, J = 8.5 Hz, 1H, Ar-**H**), 7.47 – 7.42 ($m_{apparent}$, 2H, Ar-**H**), 7.42 – 7.32 ($m_{apparent}$, 2H, Ar-**H**), 6.77 – 6.67 ($m_{apparent}$, 2H, Ar-**H**).
^{13}C{^{1}H} NMR (101 MHz, DMSO) (δ): 160.43 (d, *J* = 249.3 Hz), 159.97 (d, *J* = 248.2 Hz), 157.76 (d, *J* = 6.2 Hz), 150.26 (ddd, *J* = 248.7, 10.5, 5.1 Hz), 144.17 (t, *J* = 16.3 Hz), 142.08 (t, *J* =

11.4 Hz), 137.83 (d, *J* = 248.3 Hz), 131.32, 122.63, 120.00, 117.38, 115.07 (d, *J* = 11.9 Hz), 112.71, 112.49, 108.10 – 107.67 ($m_{apparent}$), 106.45 (t, *J* = 13.3 Hz), 103.26 (d, *J* = 24.6 Hz).
^{19}F NMR (376 MHz, DMSO) (δ): -60.40 (t, *J* = 25.7 Hz, 2F, O-C**F$_2$**-Ar), -111.61 (td, *J* = 26.5, 13.0 Hz, 2F, Ar-**F**), -115.39 (t, *J* = 11.0 Hz, 1F, Ar-**F**), -132.89 (dd, *J* = 22.0, 9.2 Hz, 2F, Ar-**F**), -163.29 (tt, *J* = 22.2, 6.0 Hz, 1F, Ar-**F**).

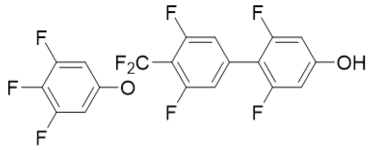

5-(2,6-difluoro-4-hydroxyphenyl)-2-(difluoro(3,4,5-trifluorophenoxy)methyl)-1,3-difluorobenzene

Yield: (off-white powder) 7.1 g, 81 %
R_f (DCM): 0.40
^{1}H NMR (400 MHz, DMSO) (δ): 10.79 (s, 1H, Ar-O**H**), 7.44 – 7.35 (m, 4H, Ar-**H**)*, 6.61 (ddd, *J* = 10.2, 2.8 Hz, 2H, Ar-**H**). (*Overlapping signals)
^{13}C{^{1}H} NMR (101 MHz, DMSO) (δ): 161.00 (dd, *J* = 246.2, 10.0 Hz), 160.41 – 159.89 ($m_{apparent}$), 157.49 (d, *J* = 5.9 Hz), 150.27 (ddd, *J* = 248.9, 10.6, 5.3 Hz), 144.50 – 143.68 ($m_{apparent}$), 137.87 (dt, *J* = 248.3, 15.3 Hz), 136.06 (t, *J* = 11.9 Hz), 122.49, 119.86, 117.48 – 116.99 ($m_{apparent}$), 115.02, 114.75, 108.02, 107.78, 105.24 (t, *J* = 18.6 Hz), 99.83, 99.56.
^{19}F NMR (376 MHz, DMSO) (δ): -60.71 (t, *J* = 26.0 Hz, 2F, O-C**F$_2$**-Ar), -111.79 (td, *J* = 26.0, 11.7 Hz, 2F, Ar-**F**), -114.54 (d, *J* = 10.8 Hz, 2F, Ar-**F**), -132.92 (dd, *J* = 22.0, 9.1 Hz, 2F, Ar-**F**), -163.29 (tt, *J* = 22.0, 6.1 Hz, 1F, Ar-**F**).

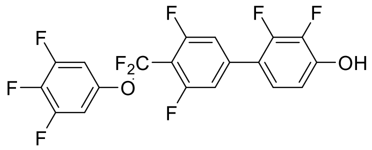

5-(2,3-difluoro-4-hydroxyphenyl)-2-(difluoro(3,4,5-trifluorophenoxy)methyl)-1,3-difluorobenzene
Yield: (white powder) 6.7 g, 76 %
R_f (DCM): 0.33
^{1}H NMR (400 MHz, DMSO) (δ): 10.90 (s, 1H, Ar-O**H**), 7.51 ($d_{apparent}$, *J* = 11.5 Hz, 2H, Ar-**H**), 7.47 – 7.37 ($m_{apparent}$, 2H, Ar-**H**), 7.32 (td, *J* = 8.7, 2.2 Hz, 1H, Ar-**H**), 6.90 (td, *J* = 8.0, 1.9 Hz, 1H, Ar-**H**).
^{13}C NMR (101 MHz, DMSO) δ 159.48 (dd, *J* = 255.2, 6.9 Hz), 151.89 (ddd, *J* = 248.0, 10.8, 5.3 Hz), 150.15 (dd, *J* = 239.6, 11.1 Hz), 148.45 (d, *J* = 6.9 Hz), 144.59 (t, *J* = 12.3 Hz), 141.54 (t, *J* = 15.0 Hz), 139.20 (d, *J* = 14.4 Hz), 137.09 (t, *J* = 15.7 Hz), 124.97 (t, *J* = 3.5 Hz), 116.89 (d, *J* = 10.6 Hz), 113.81 (t, *J* = 2.3 Hz), 113.39 (dt, *J* = 24.2, 3.4 Hz), 108.40 (dd, *J* = 23.8, 6.5 Hz), 107.53 (t, *J* = 6.2 Hz).
^{19}F NMR (376 MHz, DMSO) (δ): -60.43 (t, *J* = 25.6 Hz, 2F, O-C**F$_2$**-Ar), -111.20 (td, *J* = 25.8, 11.9 Hz, 2F, Ar-**F**), -132.84 (dd, *J* = 22.1, 9.3 Hz, 2F, Ar-**F**), -141.59 (dd, *J* = 20.5, 8.4 Hz, 1F, Ar-**F**), -160.90 (dd, *J* = 20.6, 8.0 Hz, 1F, Ar-**F**), -163.17 (tt, *J* = 22.3, 5.4 Hz, 1F, Ar-**F**).

3.2 General Esterification Protocol

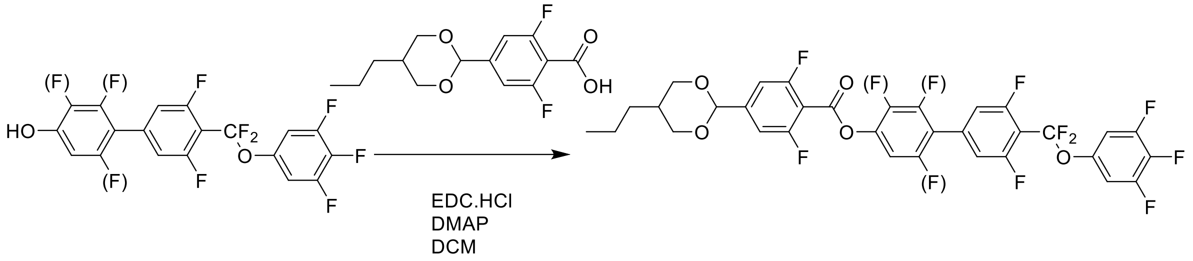

Scheme 2. General esterification protocol used in the preparation of compounds **2-4**.

The 2,6-difluoro-4-(5-propyl-1,3-dioxan-2-yl)benzoic acid (1.5 mmol), the relevant phenol (1 mmol), EDC.HCl (1.5 mmol), DMAP (5 mg) were weighed into a reaction vial or round bottom flask. DCM was added (~ 10 ml, final concentration w.r.t. phenol ~ 0.1 M), and the resulting suspension stirred until complete consumption of the starting phenol as judged by TLC. The crude reaction mixture was purified by flash chromatography using a 12 g SiO2 cartridge as the stationary phase and a gradient of hexane/DCM (0% DCM - 100% DCM) as the mobile phase, with detection made in the range 200-800 nm. The chromatographed material was filtered through a 200 nm PTFE syringe filter, concentrated to dryness, and finally recrystalised from ethanol.

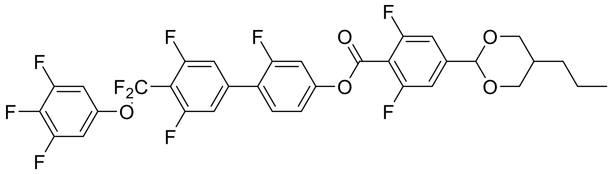

2

Yield: (white crystals) 516 mg, 75%

R_f (DCM): 0.91

^{1}H NMR (400 MHz, $CDCl_3$) (δ): 7.49 (t, J = 8.6 Hz, 1H, Ar-**H**), 7.26 – 7.16 (m, 6H, Ar-**H**)*, 7.04 – 6.95 ($m_{apparent}$, 2H, Ar-**H**), 5.40 (s, 1H, Ar-C**H**-O_2), 4.26 (dd, J = 11.8, 4.7 Hz, 2H, O-C$\mathbf{H}_{ax}$(H_{eq})-CH), 3.54 (t, J = 11.2 Hz, 2H, O-C$\mathbf{H}_{eq}$(H_{ax})-CH), 2.21 – 2.08 ($m_{apparent}$, 1H, $(CH_2)_2$-C**H**-CH_2), 1.35 (h, J = 7.6 Hz, 2H, CH_2-C$\mathbf{H_2}$-CH_3), 1.11 (q, J = 7.6 Hz, 2H, CH-C$\mathbf{H_2}$-CH_2-CH_3), 0.94 (t, J = 7.3 Hz, 3H, CH_2-C$\mathbf{H_3}$). (*Overlapping Signals)

^{13}C{^{1}H} NMR (101 MHz, $CDCl_3$) (δ): 162.21 (dd, *J* = 248.9, 6.1 Hz), 161.27 ($d_{apparent}$, *J* = 260.9 Hz), 159.56 (d, *J* = 253.7 Hz), 151.58 (d, *J* = 11.8 Hz), 151.03 (dt, *J* = 259.7, 10.9 Hz), 145.74 (t, *J* = 9.5 Hz), 144.59 (t, *J* = 14.8 Hz), 140.67 (t, *J* = 12.9 Hz), 130.62 (d, *J* = 3.7 Hz), 123.71 (d, *J* = 13.6 Hz), 120.15, 118.35 (d, *J* = 3.8 Hz), 113.14 (dt, *J* = 24.4, 3.1 Hz), 110.84 (d, *J* = 26.0 Hz), 110.33 (dd, *J* = 23.9, 2.8 Hz), 109.33 (d, *J* = 16.2 Hz), 107.48 ($m_{apparent}$), 98.79 (t, *J* = 2.2 Hz), 72.60, 33.90, 30.23, 19.53, 14.19.

^{19}F NMR (376 MHz, $CDCl_3$) (δ): -61.78 (t, *J* = 26.3 Hz, 2F, O-C$\mathbf{F_2}$-Ar), -108.41 (d, *J* = 9.6 Hz, 2F, Ar-**F**), -110.33 (td, *J* = 26.4, 11.1 Hz, 2F, Ar-**F**), -113.60 (t, *J* = 9.6 Hz, 1F, Ar-**F**), -132.43 (dd, *J* = 20.6, 8.7 Hz, 2F, Ar-**F**), -163.11 (tt, *J* = 20.8, 5.6 Hz, 1F, Ar-**F**)

HPLC (C18): 99.68 % (254 nm, 6.047 min)

HRMS (ESI +, m/z) = [m+Na]$^+$: Calculated for $C_{33}H_{22}F_{10}O_5Na$: 711.1200, found 711.1182 (error = 2.5 ppm)

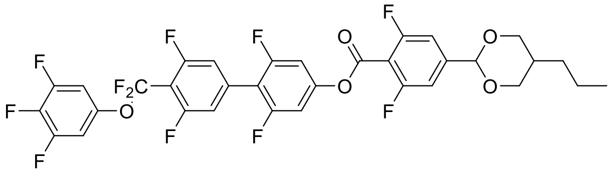

3

Yield: (white crystals) 502 mg, 71 %

R_f (DCM): 0.88

^{1}H NMR (400 MHz, $CDCl_3$) (δ): 7.20 ($d_{apparent}$, J = 10.1 Hz, 2H, Ar-**H**), 7.16 ($d_{apparent}$, J = 10.4 Hz, 2H, Ar-**H**), 7.09 – 6.94 (m, 4H, Ar-**H**)*, 5.40 (s, 1H, Ar-C**H**-O_2), 4.26 (dd, J = 11.8, 4.6 Hz, 2H, O-C$\mathbf{H}_{ax}$(H_{eq})-CH), 3.54 (t, J = 11.3 Hz, 2H, O-C$\mathbf{H}_{eq}$(H_{ax})-CH)), 2.21 – 2.07 ($m_{apparent}$, 1H, $(CH_2)_2$-C**H**-CH_2), 1.35 (h, J = 7.4 Hz, 2H, CH_2-C$\mathbf{H_2}$-CH_3), 1.11 (q, J = 7.0 Hz, 2H, CH-C$\mathbf{H_2}$-CH_2-CH_3), 0.94 (t, J = 7.3 Hz, 3H, CH_2-C$\mathbf{H_3}$). (*Overlapping Signals)

^{13}C{^{1}H} NMR (101 MHz, $CDCl_3$) (δ): 162.32 (dd, J = 258.5, 5.7 Hz), 161.00, 160.23 (dd, J = 251.6, 7.8 Hz), 158.79, 152.36 (ddd, J = 251.1, 10.3, 5.6 Hz), 151.38 (t, J = 14.3 Hz), 146.05 (t, J = 9.7 Hz), 139.76 (t, J = 14.0 Hz), 134.28 (t, J = 10.0 Hz), 122.68, 120.02, 117.38, 114.76 (d, J = 24.7 Hz), 110.36 (dd, J = 23.6, 3.5 Hz), 108.91, 107.75 – 107.29 ($m_{apparernt}$), 106.97 – 106.47 ($m_{apparent}$), 98.73 (t, J = 2.0 Hz), 72.60, 33.90, 30.22, 19.53, 14.18.

^{19}F NMR (376 MHz, $CDCl_3$) (δ): -61.95 (t, *J* = 26.5 Hz, 2F, O-C$\mathbf{F_2}$-Ar), -108.15 (d, *J* = 10.0 Hz, 2F, Ar-**F**), -110.53 (td, *J* = 26.5, 10.7 Hz, 2F, Ar-**F**), -111.71 (d, *J* = 9.1 Hz, 2F, Ar-**F**), -132.43 (dd, *J* = 20.9, 8.6 Hz, 2F, Ar-**F**), -163.07 (tt, *J* = 20.7, 5.9 Hz, 1F, Ar-**F**).

HPLC (C18): 99.58 % (254 nm, 6.060 min)

HRMS (ESI +, m/z) = [m+Na]$^+$: Calculated for $C_{33}H_{21}F_{11}O_5Na$: 729.1106, found 729.1101 (error = 0.7 ppm)

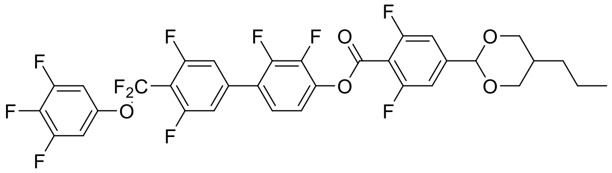

4

Yield: (white crystals) 435 mg, 62 %

R_f (DCM): 0.90

^{1}H NMR (400 MHz, $CDCl_3$) (δ): 7.29 – 7.16 (m, 6H, Ar-**H**)*†, 7.00 (dd, *J* = 7.9, 5.8 Hz, 2H, Ar-**H**), 5.41 (s, 1H, Ar-C**H**-O_2), 4.26 (dd, *J* = 11.7, 4.6 Hz, 2H, O-C$\mathbf{H}_{ax}$(H_{eq})-CH), 3.54 (t, *J* = 11.4 Hz, 2H, O-C$\mathbf{H}_{eq}$(H_{ax})-CH)), 2.21 – 2.07 ($m_{apparent}$, 1H, $(CH_2)_2$-C**H**-CH_2), 1.35 (h, *J* = 7.7 Hz, 2H, CH_2-C$\mathbf{H_2}$-CH_3), 1.11 (q, *J* = 7.1 Hz, 2H, CH-C$\mathbf{H_2}$-CH_2-CH_3), 0.94 (t, *J* = 7.3 Hz, 3H, CH_2-C$\mathbf{H_3}$). (*Overlapping peaks, †Overlapping $CDCl_3$ peak)

^{13}C{^{1}H} NMR (101 MHz, $CDCl_3$) (δ): 162.43 (dd, *J* = 259.8, 5.4 Hz), 161.34 (dd, *J* = 259.2, 6.3 Hz), 158.22, 150.82 (ddd, *J* = 250.5, 10.6, 5.1 Hz), 150.03, 147.45 (dd, *J* = 245.7, 12.0 Hz), 146.18 (t, *J* = 9.9 Hz), 144.85 – 144.33 ($m_{apparent}$), 142.49 (d, *J* = 15.0 Hz), 140.04 – 139.30 ($m_{apparent}$), 137.35 (t, *J* = 15.1 Hz), 125.49 (d, *J* = 9.5 Hz), 123.73 (t, *J* = 4.0 Hz), 122.69, 120.04, 119.15 (d, *J* = 4.0 Hz), 113.14 (dt, *J* = 24.7, 3.3 Hz), 110.38 (dd, *J* = 23.5, 3.5 Hz), 109.58 (t,

J = 14.3 Hz), 108.43 (t, *J* = 16.8 Hz), 107.80 – 107.30 ($m_{apparent}$), 98.73 (t, *J* = 1.6 Hz), 72.58, 33.90, 30.22, 19.53, 14.16.
^{19}F NMR (376 MHz, $CDCl_3$) (δ): -61.87 (t, *J* = 26.4 Hz, 2F, O-C**F₂**-Ar), -107.59 (d, *J* = 10.0 Hz, 2F, Ar-**F**), -109.76 (td, *J* = 26.5, 10.7 Hz, 2F, Ar-**F**), -132.40 (dd, *J* = 20.7, 8.7 Hz, 2F, Ar-**F**), -138.63 (dd, *J* = 20.1, 6.5 Hz, 1F, Ar-**F**), -147.62 (dd, *J* = 20.3, 5.6 Hz, 1F, Ar-**F**), -163.04 (tt, *J* = 21.0, 5.0 Hz, 1F, Ar-**F**).
HPLC (C18): 99.81 % (254 nm, 6.061 min)
HRMS (ESI +, m/z) = $[m+Na]^+$: Calculated for $C_{33}H_{21}F_{11}O_5Na$: 729.1106, found 729.1101 (error = 0.6 ppm)

3.3 Example NMR Spectra, HPLC Chromatograms, and HRMS Spectra.

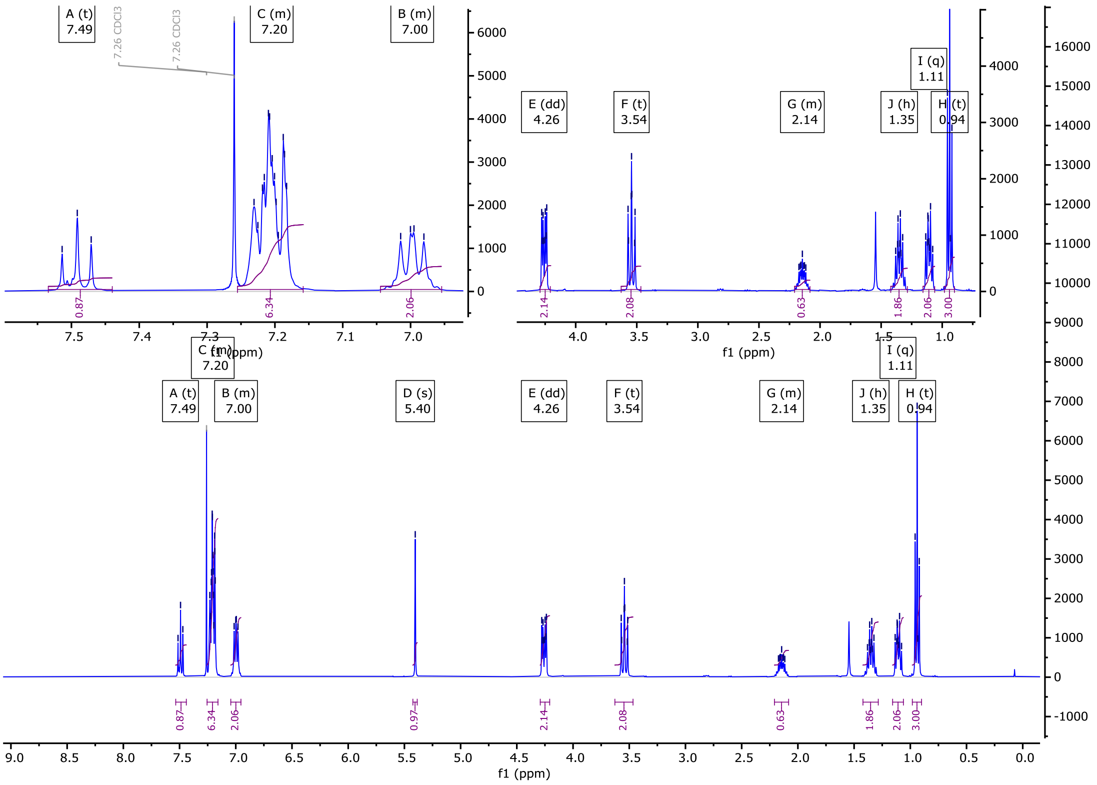

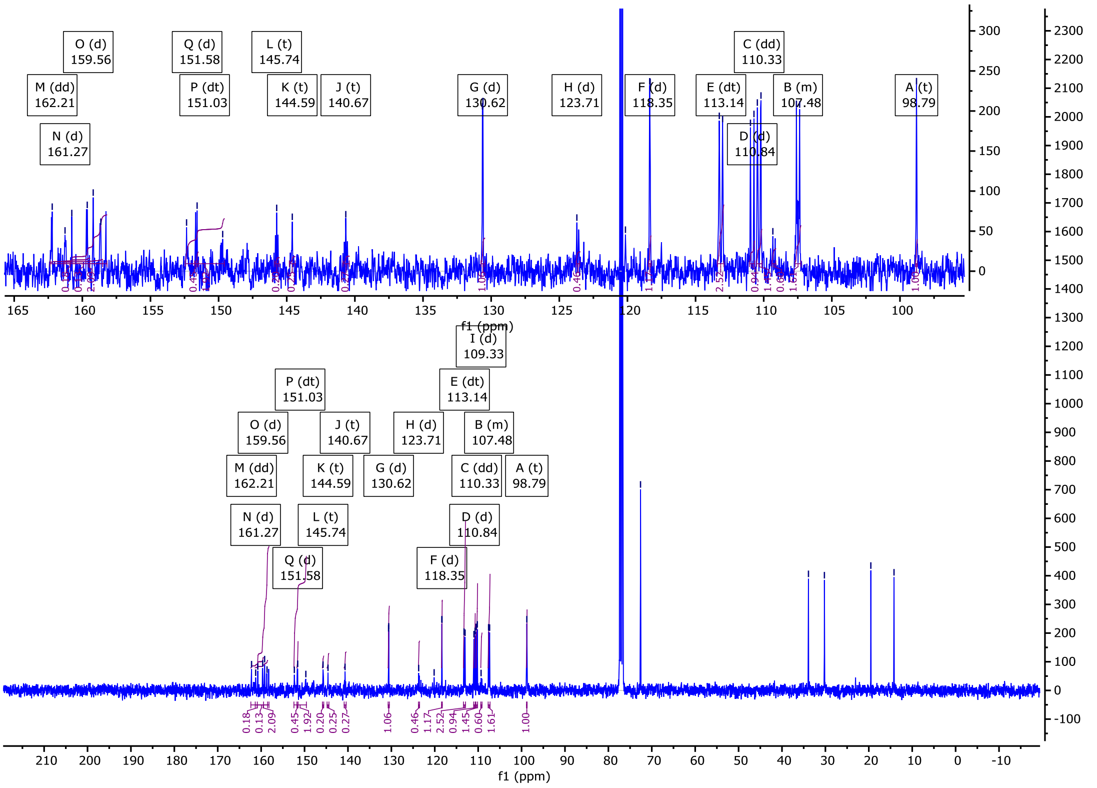

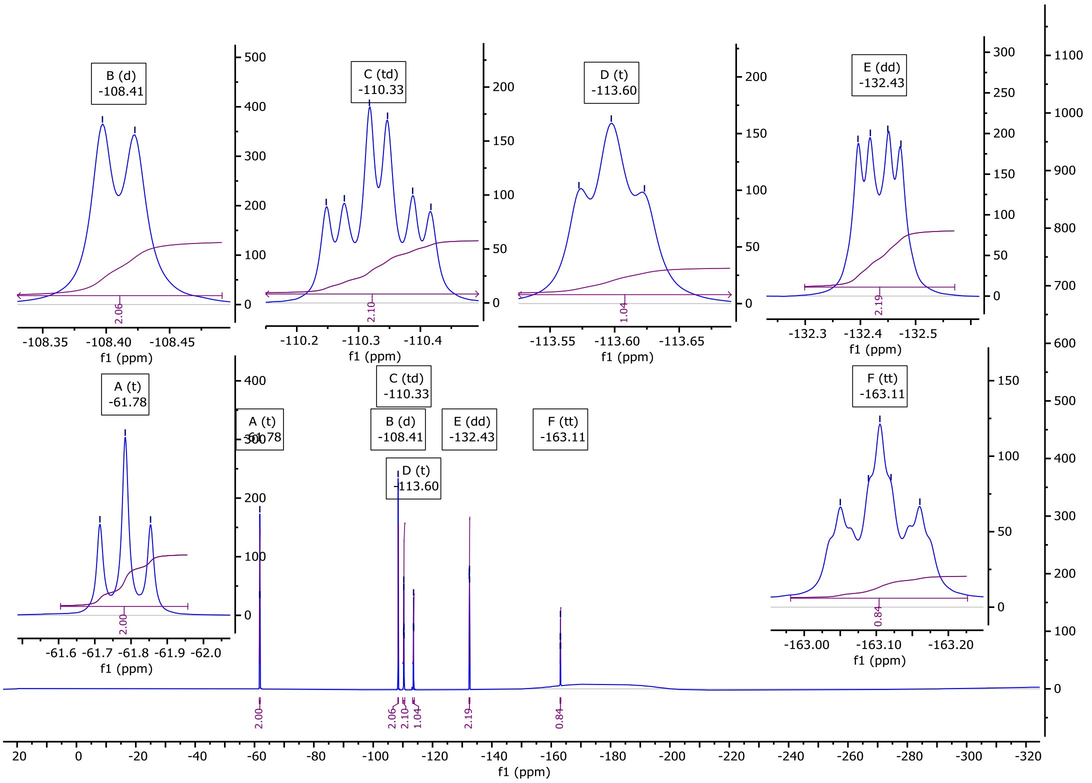

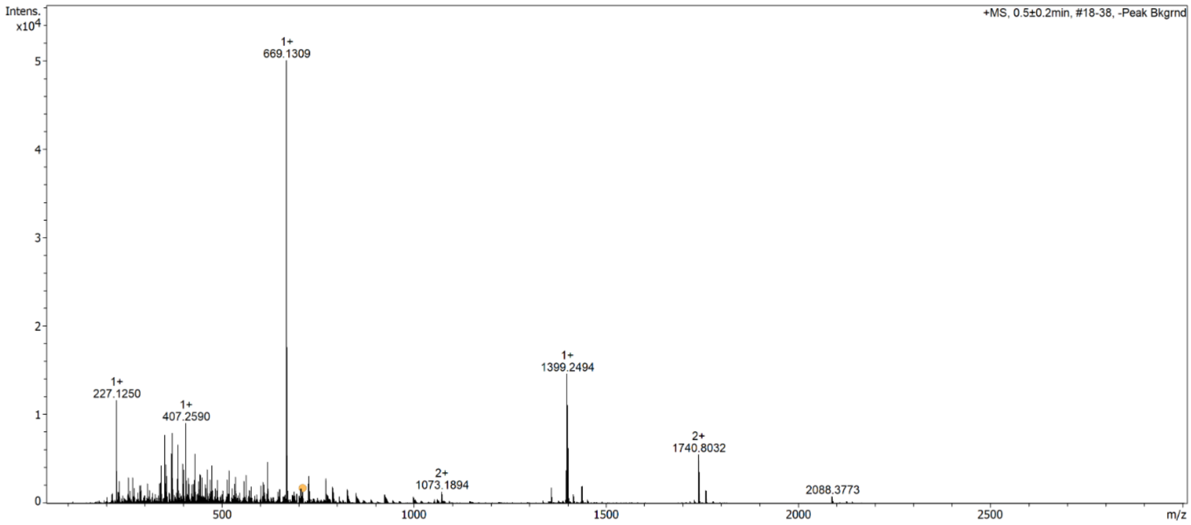

Fig. S14. ^{1}H [top], ^{13}C{^{1}H} [middle], and ^{19}F [bottom] NMR spectra, and HRMS Spectra for **2**.

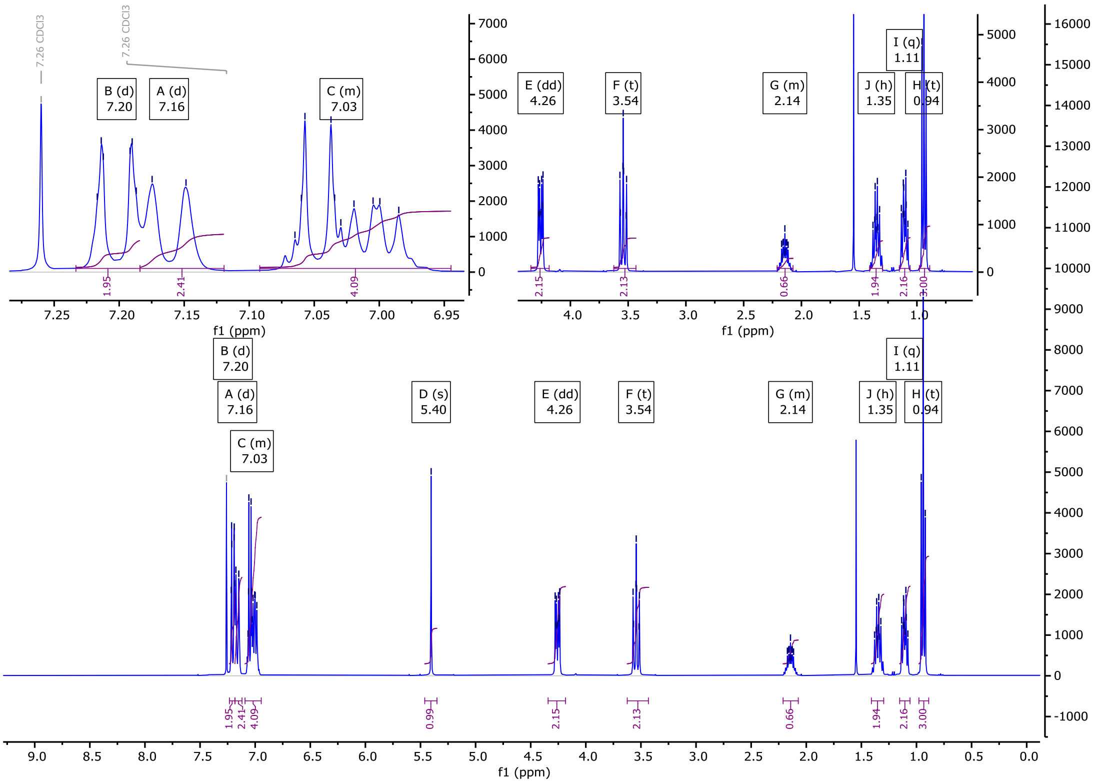
7.26 CDCl3
B (d) 7.20
A (d) 7.16
C (m) 7.03
E (dd) 4.26
F (t) 3.54
G (m) 2.14
I (q) 1.11
J (h) 1.35
H (t) 0.94
D (s) 5.40
1.95
2.41
4.09
0.99
2.15
2.13
0.66
1.94
2.16
3.00
f1 (ppm)

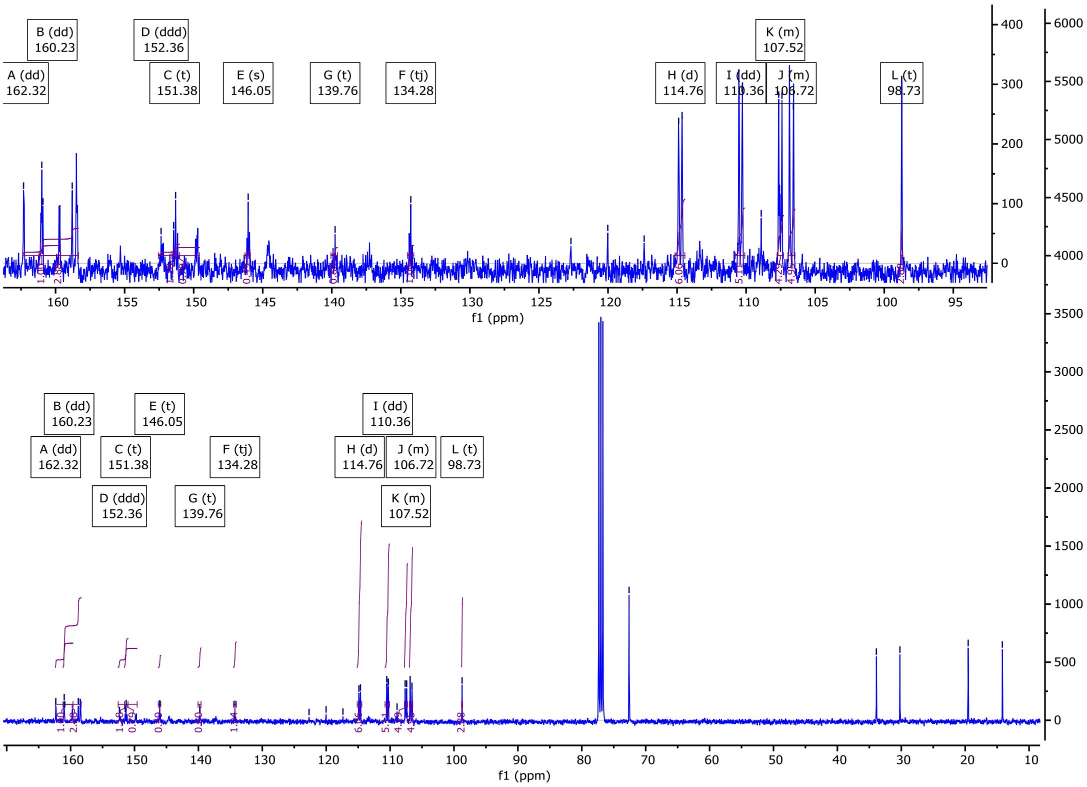
B (dd) 160.23
A (dd) 162.32
D (ddd) 152.36
C (t) 151.38
E (s) 146.05
G (t) 139.76
F (tj) 134.28
H (d) 114.76
I (dd) 110.36
K (m) 107.52
J (m) 106.72
L (t) 98.73
E (t) 146.05
f1 (ppm)

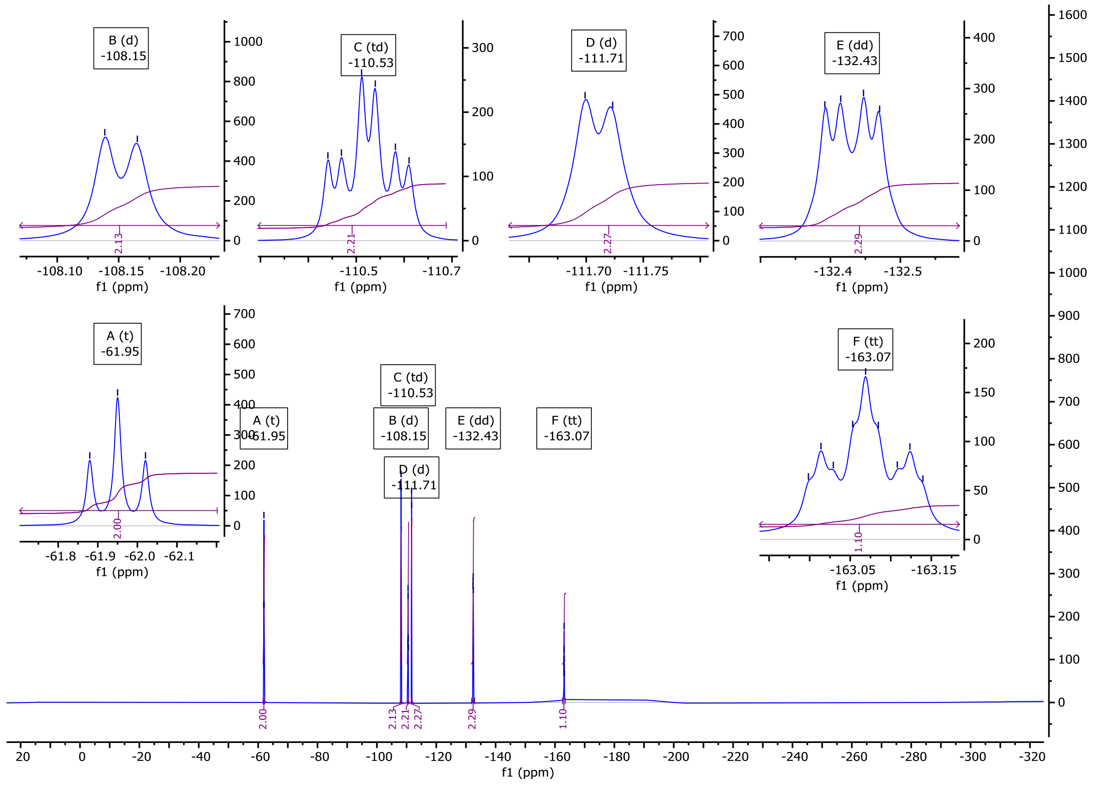

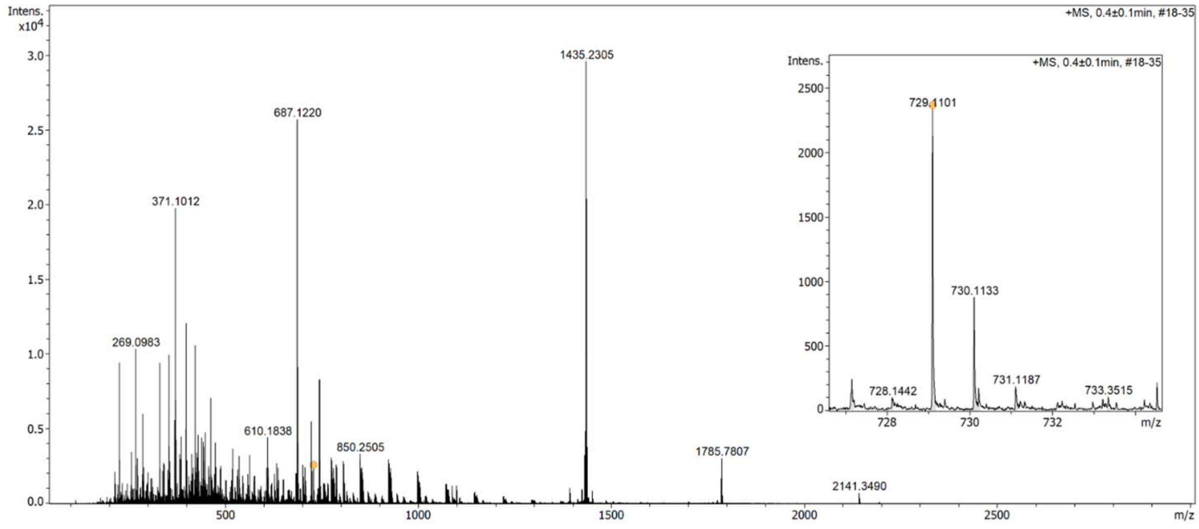

Fig. S15. ^{1}H [top], $^{13}C\{^{1}H\}$ [middle], and ^{19}F [bottom] NMR spectra, and HRMS Spectra for **3**.

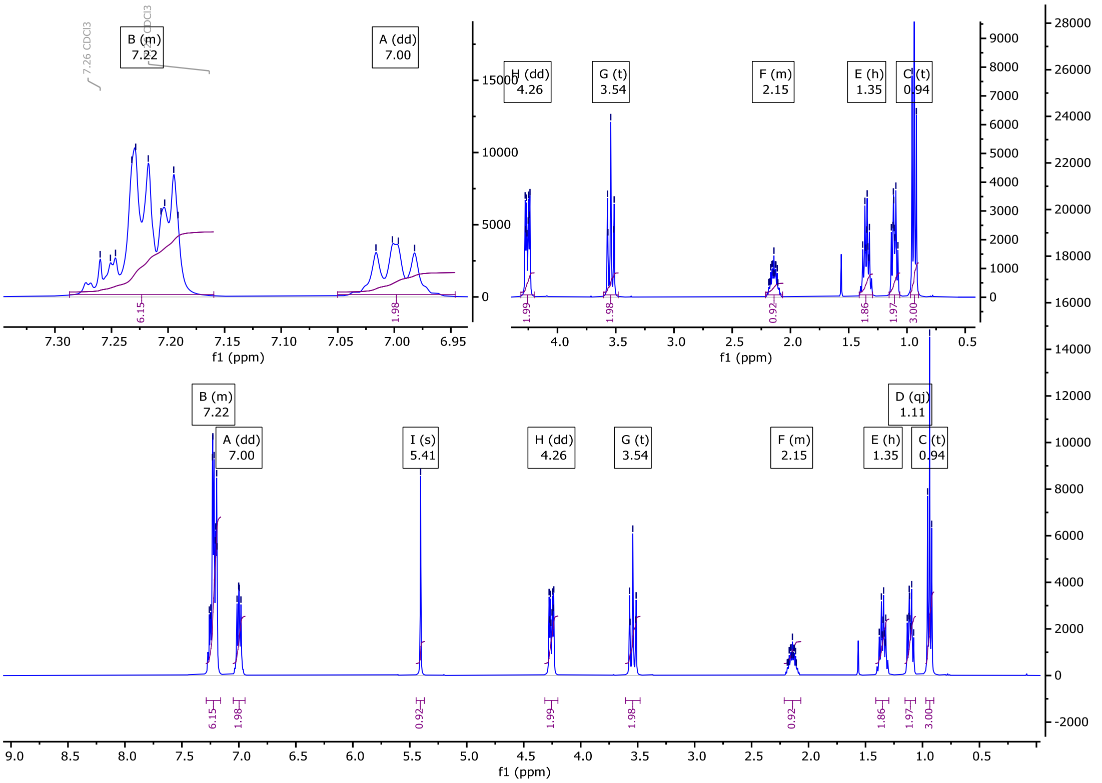

7.26 CDCl3
B (m) 7.22
A (dd) 7.00
H (dd) 4.26
G (t) 3.54
F (m) 2.15
E (h) 1.35
C (t) 0.94
D (qj) 1.11
I (s) 5.41
f1 (ppm)

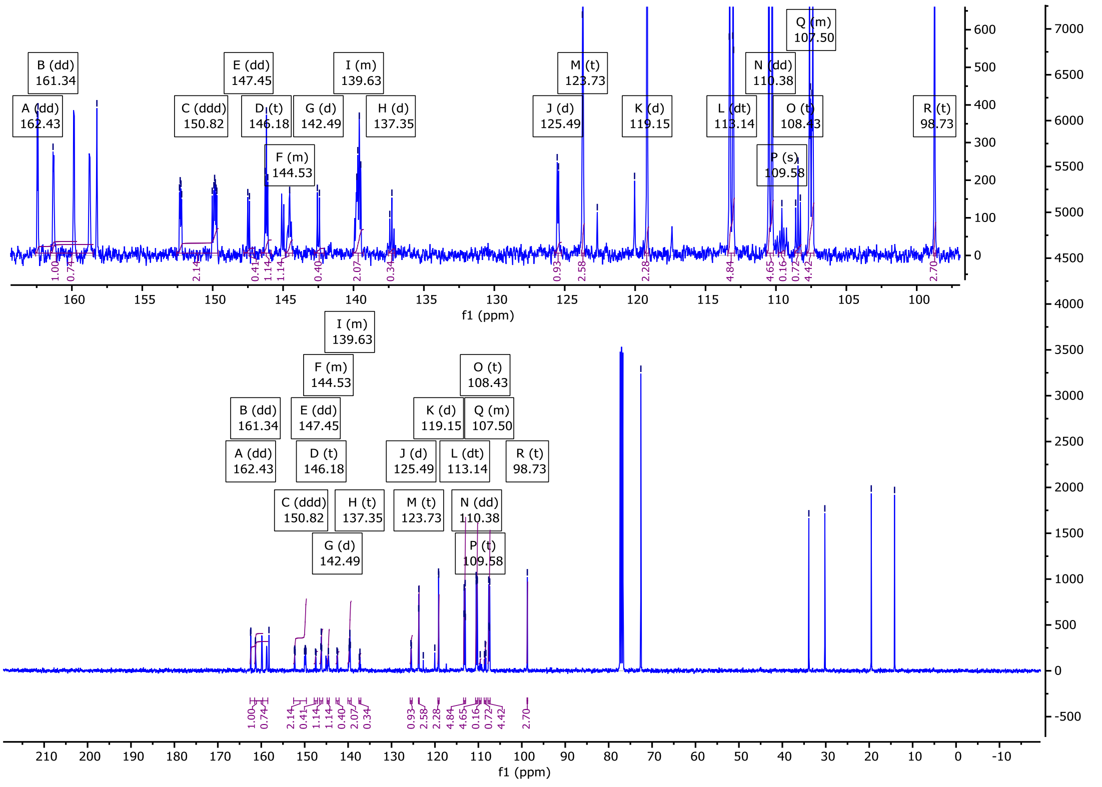

A (dd) 162.43
B (dd) 161.34
C (ddd) 150.82
D (t) 146.18
E (dd) 147.45
F (m) 144.53
G (d) 142.49
H (d) 137.35
I (m) 139.63
J (d) 125.49
K (d) 119.15
L (dt) 113.14
M (t) 123.73
N (dd) 110.38
O (t) 108.43
P (s) 109.58
Q (m) 107.50
R (t) 98.73
f1 (ppm)

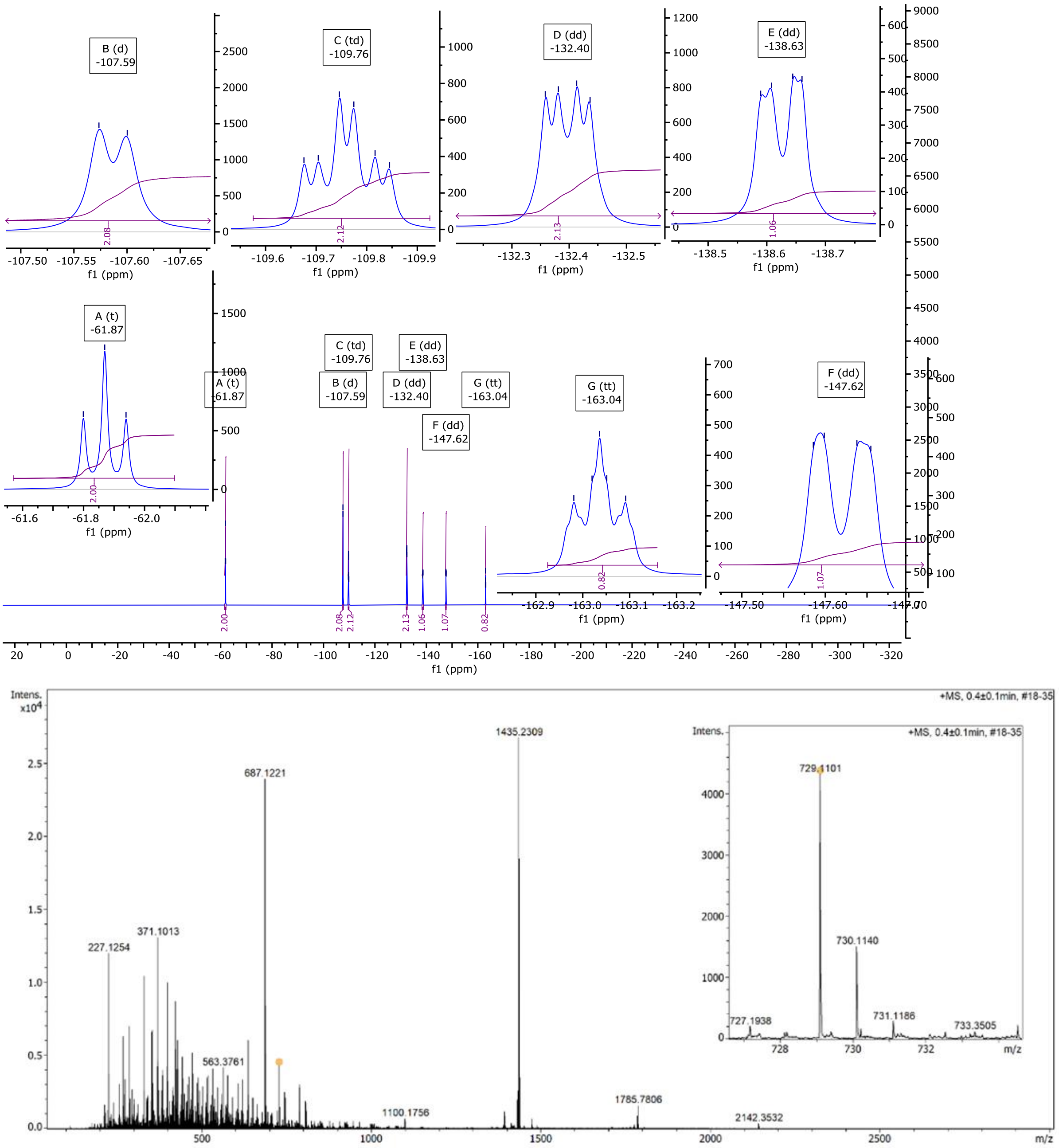

Fig. S16. ^{1}H [top], $^{13}C\{^{1}H\}$ [middle], and ^{19}F [bottom] NMR spectra, and HRMS Spectra for **4**.

4. Supplemental references

[1] Gibb, C.J., Hobbs, J., Nikolova, D.I. et al. Spontaneous symmetry breaking in polar fluids. Nat Commun, 15, 5845 (2024)

[2] Hobbs, J., Gibb, C.J. and Mandle, R.J. Emergent Antiferroelectric Ordering and the Coupling of Liquid Crystalline and Polar Order. Small Sci., 4, 2400189 (2024)
