# Supplementary material for: Ferri- and ferro-electric switching in spontaneously chiral polar liquid crystals
Source: Nat Commun. 2025 Aug 13;16:7510. doi: 10.1038/s41467-025-62684-z (PMC12350638; doi:10.1038/s41467-025-62684-z)
Supplement: Supplementary file 2 — Description of Additional Supplementary Files [file 41467_2025_62684_MOESM2_ESM.pdf]

File Name: Supplementary Video 1

Description: Video of light diffraction by sample 3 as it transitions on cooling between the N_F phase and the SmC_P^H phase.

File Name: Supplementary Video 2

Description: Video of light diffraction by sample 3 as it transitions on heating between the SmC_P^H phase and the N_F phase.
